# Three Months-Old’ Preferences for Biological Motion Configuration and Its Subsequent Decline

**DOI:** 10.3390/brainsci12050566

**Published:** 2022-04-27

**Authors:** Isabel C. Lisboa, Daniel M. Basso, Jorge A. Santos, Alfredo F. Pereira

**Affiliations:** 1 Psychology Research Centre (CiPsi), School of Psychology, Campus de Gualtar, University of Minho, 4710-057 Braga, Portugal; 2 Algoritmi Research Centre, School of Engineering, Campus de Azurém, University of Minho, 4800-058 Guimarães, Portugal; jorge.a.santos@psi.uminho.pt; 3 UNINOVA-CTS, Campus de Caparica, NOVA University of Lisbon, 2829-516 Caparica, Portugal; dmb@uninova.pt (D.M.B.); ap@uninova.pt (A.F.P.); 4 Centre for Computer Graphics, 4800-058 Guimarães, Portugal; 5 School of Psychology, Campus de Gualtar, University of Minho, 4710-057 Braga, Portugal

**Keywords:** infancy, action understanding, biological motion, preferential looking, configural processing, point-light walkers

## Abstract

To perceive, identify and understand the action of others, it is essential to perceptually organize individual and local moving body parts (such as limbs) into the whole configuration of a human body in action. Configural processing—processing the relations among features or parts of a stimulus—is a fundamental ability in the perception of several important social stimuli, such as faces or biological motion. Despite this, we know very little about how human infants develop the ability to perceive and prefer configural relations in biological motion. We present two preferential looking experiments (one cross-sectional and one longitudinal) measuring infants’ preferential attention between a coherent motion configuration of a person walking vs. a scrambled point-light walker (i.e., a stimulus in which all configural relations were removed, thus, in which the perception of a person is impossible). We found that three-month-old infants prefer a coherent point-light walker in relation to a scrambled display, but both five- and seven-month-old infants do not show any preference. We discuss our findings in terms of the different perceptual, attentional, motor, and brain processes available at each age group, and how they dynamically interact with selective attention toward the coherent and socially relevant motion of a person walking during our first year of life.

## 1. Introduction

Early perceptual and attentional biases are a fundamental constraint in socio-cognitive development. By actively preferring and sustaining attention to social stimuli, infants co-create with other social partners the vital input for their development [1]. This is especially important in the first months of life, where infants depend on caregivers for all of their allostatic needs [1], and reaching, grasping, and self-initiated locomotion are absent or at best immature [2]. Across several classes of stimuli with high social relevance, including speech [3,4], faces [5], and biological motion [6,7], human newborns show innate sensitivities—e.g., spend more time looking at certain stimuli than other. While the exact source of these constraints at birth continues to be under debate, it is clear that the large developmental changes observed in the first year in the processing of social stimuli cannot be explained solely by a maturational program that is triggered by experience [1,8,9,10].

The case of face perception is revealing, in itself, and for other domains of perceptual stimuli that populate infants’ environment [11,12]. The specialization in face perception, that starts in the first three months of life, is experience expectant [13,14], and it leads to a major developmental milestone: an emergent sensitivity to the configural properties of faces [5]. For example, in contrast with newborns [15], three-month-old infants will prefer to look at a face when contrasted with a top-heavy scrambled version of the same face [5,15]. The control stimulus in this study was created by rearranging the facial features, disrupting the structure of the face, and in a way that maximized the top-heavy property, known to be preferred by newborns (i.e., more elements in the upper part). This example contains a few important aspects, the first being that it shows that older infants can engage in configural processing of a face (i.e., processing the relations among features or parts), a finding corroborated by the extant evidence from face-perception studies. This is a more sophisticated form of visual perception, where the structural properties of an object are taken into consideration (in faces, for instance, the arrangement of eyes, nose, and mouth) as opposed to the mere presence of local featural properties (in faces, the presence of eyes, irrespective of their location, orientation, and relations with other parts). Of significance, this example also demonstrates that in the domain of face perception, at least by three months of age, the processing of configural information contributes to selective visual attention. In effect, configural processing influences preferential attention and acts as a developmental constraint, thereby contributing to the early specialization in face perception and likely also to the impressive expertise in face perception observable later in development.

In the present work, motivated by the same reasoning, we are concerned with another domain in visual perception, biological motion, and with the question of when, in early infancy, the presence of biological motion configuration (the configural information within a moving body) leads to preferential attention toward it: that is, when do infants prefer biological motion configuration, and not just the mere presence of local featural information?

In two experiments, we measured infants’ preference for biological motion configuration after birth. Participants were tested in a preferential looking procedure, contrasting an approaching walking person, with a scrambled version of the same walking motion that preserved featural information. To the best of our knowledge, no previous study has tested this comparison using the preferential looking task, with the exception of studies with neonates [6,7]. We hypothesize that infants prefer a stimulus that corresponds to the coherent and socially relevant motion of a person walking, instead of a stimulus with no coherent configuration.

### 1.1. Background

#### 1.1.1. When Infants Begin to Perceive Configural Relations in Biological Motion

Biological motion is a term typically used to designate the motion patterns of living organisms [16]. The perception of biological motion has been extensively studied with the use of point-light displays, a type of controlled visual stimulus where only a few dots, placed on the major joints of a moving person, are visible. Studies of biological motion configuration typically use the comparison of a coherent intact point-light walker (i.e., a point-light display of the walking action) with a spatially scrambled point-light walker. The scrambled control is a standard manipulation from visual perception studies, and it consists of randomizing the positions of the individual elements that compose the visual stimulus. In the case of biological motion, the initial positions of the dots of a point-light walker are scrambled or randomized. As a result, the scrambled point-light walker has the same set of individual moving dots as the original display (with each dot’s individual trajectory preserved) but with all configural relations disrupted—perceptual grouping or configural processing of the dots is impossible, thus, there is neither the possibility of perceiving a human body, nor moving limbs.

In a series of infant-controlled habituation experiments, Bertenthal and colleagues found that the human capacity to perceive configural relations in biological motion emerges in infancy, developing gradually from three to five months of age [17]. Infants at three months are more tuned to local configural information (i.e., to the relation between sets of dots in a display, for example, to the relation between the three dots placed over the shoulder, elbow, and wrist that compose a moving arm [17]). Only by five months, infants begin to perceive the relation between all dots in the display (i.e., the global configuration of the human walking action [17]). Both three- and five-month-old discriminate a coherence from a scrambled point-light walker because infants at both ages process at least some level of configural information in point-light walkers [18,19]. However, only five-month-old discriminate point-light stimuli at a more mature global configural level. For example, only five-month-old discriminate an intact point-light walker from a divided point-light walker [20,21]. A divided point-light walker consists of a point-light walker cut at the waist level, into a top and a bottom half with the two halves presented simultaneously, horizontally separated [20,21]. This control stimulus has equal and preserved local configural information, thus, three-month-old infants do not discriminate between the two stimuli. Discrimination in this case necessarily implies global configural processing—and only five-month-old infants discriminate an intact from a divided point-light walker.

A more recent habituation experiment confirmed these conclusions by reporting that six- and nine-month-old infants perceive human point-light walkers as solid forms, that cannot pass through a table [22]. In this experiment, infants associated a solid global form only with the intact point-light walker and not with a scrambled or an inverted walker [22].

Finally, neurodevelopmental studies also show early brain-processing differences between scrambled and coherent point-light displays at eight [23], seven [24,25], and five months of age [26]. In particular, Lisboa and Queirós et al. (2020) used functional near-infrared spectroscopy (fNIRS) and found brain processing differences in infants’ right superior temporal sulcus (STS) region—the same region associated with adults’ cortical processing of biological motion configuration [27,28,29,30,31,32]. This result indicates that, at least at seven months, infants show mature-like processing of intact point-light walkers vs. scrambled controls, and that the perception of configural relations involves the functioning of a high-level cortical region at this age.

In summary, both habituation and neuroimaging experiments indicate that infants at three months process local configural information and only later, by five to seven months of age, the global configuration of the walking action. However, none of these studies investigated if infants actively seek and prefer to look at biological motion configuration.

#### 1.1.2. Preferential Attention to Biological Motion in the First Year

Infants prefer to look at biological motion since the very moment they are born [6,7]. Studies with newborns show that typically developing two-day-old infants spend more time or prefer the point-light walker of a chicken in relation to rigid object motion [6], and to an inverted point-light walker [7]. Nevertheless, preferences for biological motion are still limited at birth [33,34]: when contrasting a coherent with a scrambled point-light walker, newborns show no preference for either stimulus [6]. Newborns equally turn at a scrambled and a coherent point-light walker, indicating that humans at birth are unable to prefer configural relations in biological motion [6]. Despite this result, few studies investigated preferences for biological motion after birth, and none contrasting a coherent to a scrambled display.

Fox and McDaniel (1982) conducted the seminal preference study. They concluded that some level of visual experience is needed for infants to prefer biological motion: two-month-old infants show no preferences between an intact point-light walker and a set of moving dots; both four- and six-month-old infants prefer an upright in relation to an inverted point-light walker; and only six-month-old prefer an on-joint display of a hand moving over the same display with off-joint markers [35].

More recently, Sifre et al. (2018) tested infants longitudinally, from 2 to 24 months of age, in a preferential looking task with upright and inverted point-light displays. They used intact upright point-light displays of an actor playing children’s games (e.g., “peek-a-boo”), and inverted versions of these displays. As in the Fox and McDaniel (1982) experiment, two-month-old infants showed no preferences. A preference for the upright displays emerged at three months, and infants showed an increased preference for the upright displays across developmental age, that is, the older the infant, the longer he/she tended to look at intact displays [34].

Common to these two preferential looking experiments [34,35] is infants’ increased sensitivity and preference for biological motion with age. However, next, we outline important research gaps in these studies, that remain to be investigated; these gaps also motivated the current experiments.

First, Fox and McDaniel (1982) and Sifre et al. (2018) found no preferences for intact point-light displays in infants aged two months—but these results seem to be in contradiction with the newborn findings (that report preferential attention to intact point-light walkers over rigid and inverted displays [7,33]). In fact, these newborn studies report that infants show no preference between a coherent and a scrambled point-light walker at birth [6]—no other study investigated when, in infancy, a preference for biological motion configuration emerges. Second, both Fox and McDaniel (1982) and Sifre et al. (2018) employed an inverted control—a preference for an upright over an inverted display might simply be explained by a sensitivity in infancy to the gravity-dependent dynamics of motion, which is not specific to human motion or to the perception of biological motion. Finally, concluding about the role of biological motion configuration in defining infants’ preferences is problematic: the two studies compared an intact with an inverted point-light display; inverted stimuli still depict coherent local and global configural relations between the dots. The scrambled manipulation is a classical control stimulus from visual perception and biological motion studies that specifically addresses the role of configural information; furthermore, the use of this control stimulus correctly responds to all of the referred limitations.

### 1.2. Current Experiments: When Do Infants Prefer Biological Motion Configuration?

We present two experiments (one cross-sectional and one longitudinal) measuring preferential attention to biological motion configuration after birth. Three- to seven-month-old infants were tested in two preferential looking experiments contrasting a coherent to a scrambled point-light walker approaching the viewer; we measured the amount of time and the proportion of looking at the intact coherent point-light walker to determine a preference.

In the first experiment (Experiment 1, cross-sectional), we tested two groups of infants aged three and five months. In the second experiment (Experiment 2, longitudinal), we asked parents that participated with their infants at three months in Experiment 1 to come again both at five and seven months of age. We hypothesize that, because the ability to process configural relations in human motion emerges in infancy, particularly at around three to five months of age [17], a preference for a coherent point-light walker in relation to a scrambled walker should emerge around this developmental period. Moreover, previous experiments found preferential attention to upright biological motion emerging at three months and increasing with age [34,35]. Infants turn their attention to important stimuli in their environments; we reason that, if infants are able to process the meaningful and coherent configuration of a person walking (or at least some level of configural information such as the motion of limbs), they might prefer a coherent point-light walker in relation to a scrambled control, that is, a stimulus with no configural information.2. 

## 2. Experiment 1: Cross-Sectional Study with 3- and 5-Month-Old Infants

### 2.1. Method

#### 2.1.1. Participants

Fifty infants (*n* = 50) took part in the experiment: *n* = 27 three-month-old infants (10 females, M = 104.3 days, SD = 10.9, age range = 89.0—128.0 days) and *n* = 23, five-month-old infants (13 females, M = 166.6 days, SD = 10.3, age range = 143.0—188.0 days).

At three months of age, only data from *n* = 17 infants were processed (37.0% attrition rate): one infant was excluded due to technical problems, three due to fussiness, and six did not pass the two looking criteria. More details on the looking criteria are in the Data coding and processing section. At five months of age, the final sample comprised *n* = 17 infants (26.09% of attrition rate): two infants were eliminated due to technical problems, and four infants did not pass the looking criteria. In sum, the final sample of Experiment 1 consisted of *n* = 34 infants, half with three months and half with five months of age.

All infants were born full-term (at least 37 weeks of gestation and birth weight > 2500 g) and had no family history of neurological disorders or reported hearing or vision problems.

Infants and parents were recruited from pediatrician clinics, public health centers, and pregnancy gymnastic classes. All parents gave their written informed consent before participating. The University of Minho ethics committee approved the study.

#### 2.1.2. Stimuli

Two motion stimuli were used in this study: (1) an intact coherent point-light walker, and (2) a spatially scrambled version of this display.

The coherent point-light walker was captured using a VICON motion capture system at 240 Hz. It consisted of 13 white spheres placed on the major joints (head, right and left shoulders, hips, ankles, knees, wrists, and elbows) of a female model walking against a black background [24,25,36]. The 13 spheres measured each 0.68° of visual angle at 50 cm of visual distance. The point-light walker described a frontal approaching motion (1.34 m/s), walking two steps or one complete step cycle (2 s) as if on a treadmill, (i.e., with the translational displacement removed). The stimulus measured 21.31 in height and 8.53º in width at a visual distance of 50 cm.

The scrambled point-light walker was created by randomizing the initial position of the 13 dots that composed the coherent point-light walker, inside of a bounding box with approximately the same width and height as the original coherent point-light walker. Thus, both stimuli (coherent and scrambled) had the same duration and an equivalent angular size (the scrambled point-light walker measured 22.29° in height and 7.97° in width). The scrambled point-light walker consisted of an elongated random displacement of the dots, where all spatial–temporal relations between the dots were disrupted. Both local (the spatial–temporal relation between at least two dots) and global (the spatial–temporal relation between all dots that compose the coherent display that corresponds to the form of a person walking) configural relations were perturbed in the scrambled display. Despite this, the number, size, luminance and velocity profile of the individual moving dots were the same in both stimuli. The individual motion of each dot was also kept intact in the scrambled point-light walker.

The two point-light-walkers were animated in Blender, an open-source 3D graphics program [37].

#### 2.1.3. Procedure

Preference between the two motion stimuli (coherent and scrambled) was assessed by a preferential looking procedure.

The main apparatus consisted of two side-by-side identical computer screens (6.5 cm apart, screen size: 53 cm × 30 cm, ASUS VG248QE monitor, with a resolution of 1920 × 1080, 144 Hz) placed in a room with no windows and illuminated only by a dim light located behind and in the middle of the two screens. The screens were framed with black cardboard, and a webcam (model HP HD 4310, 30 Hz) was placed in the middle of the screens only for online tracking of the infants’ behavior during the experiment. Infants’ looking behavior during stimuli presentation was recorded by a second high-definition video camera (Panasonic HC-V777, sampling frequency of 50 Hz) also placed in the middle of the screens. Control of the experiment (starting a new trial, rendering the stimuli, synchronization with the camera recording) was achieved using custom-made software. The two point-light walker stimuli were presented, one on each computer monitor, at a resolution of 1920 × 1080 pixels, and rendered in real-time using Blender 2.78 [37] and at 60 Hz.

During the experiment, infants sat on their parent’s laps, 50 cm away from the middle of the two screens. Parents were instructed to (1) refrain from interacting with the infant—they were specifically told to not interact with the infant and only smile if the baby searched for their face; (2) maintain the infant centered on his/her lap; and, finally, (3) not to turn their own body or head to either side of the screens and to fixate on the middle of the two screens.

**Figure 1:**
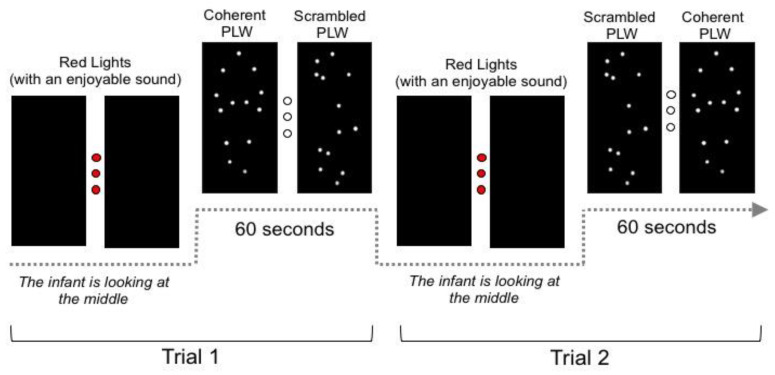
A schematic representation of the procedure in Experiment 1. Red lights were displayed at the beginning of each trial to attract infant’s attention to the middle of the screens. Only then, the experimenter triggered the beginning of the stimuli, and the coherent and scrambled point-light walkers (PLWs) were presented simultaneously for 60 s. Infants participated in a total of 6 trials.

Once the infant was calm and seemed ready, the experiment started. Each trial was structured as follows: the apparatus started by displaying three small red lights, equidistant from the two stimuli, in sync with an enjoyable toy sound, to attract infants’ attention to the middle of the two screens. Once the infant looked at the lights (i.e., to the middle), the experimenter triggered the beginning of the stimuli (the experimenter tracked the infant’s looking using a webcam)—see Figure 1 for a schematic representation of the procedure. The actual experimental period consisted of presenting the two motion stimuli simultaneously, one per screen [33,38]. The two stimuli were separated by 46.6 cm horizontally (42.98° at 50 cm of visual distance), and a left/right position for the coherent and scrambled point-light walker was counterbalanced across trials per individual subject; the order of the trials was also randomized per subject. Infants participated in a total of 6 trials with a 60 s duration each [33,34]. To reach 60 s of trial duration, the point-light walkers were looped, thus, each step cycle (2 s of duration) was looped 30 times. The experiment lasted approximately 6 min. If the infant grew restless or upset, the experiment ended earlier.

#### 2.1.4. Data Coding and Processing

To be included on the final sample, infants had to (1) show sufficient looking (i.e., at least 50% of the time looking at any screen [35]) and (2) not show any side bias (i.e., have at least 10% of looking time at each screen [33,39]).

A trained human coder, unaware of the experimental design or of which stimuli were on each screen, coded the infants’ eye fixations. Coding was conducted on a frame-by-frame basis using Blender 2.78, an open-source 3D modeling software [37]. Infants’ fixations were classified according to three categories: (1) looking at the right stimulus from the midline of the screens; (2) looking at the left stimulus from the midline of the screens; and (3) looking elsewhere.

The looking time toward the coherent point-light walker was calculated per trial and transformed into a proportion by dividing the amount of time spent looking at the coherent point-light walker by total time spent looking at the scrambled or coherent point-light walker. This proportion of looking at the coherent point-light walker per trial was then averaged per subject. The distribution of the proportion of time looking at the coherent point-light walker, in both age groups, followed a normal distribution (confirmed by visual inspection and a Shapiro–Wilk test) and there was homogeneity of variances; hence, we used parametric statistics. All analyses and transformations to the data were done in R [40].

### 2.2. Results

**Figure 2:**
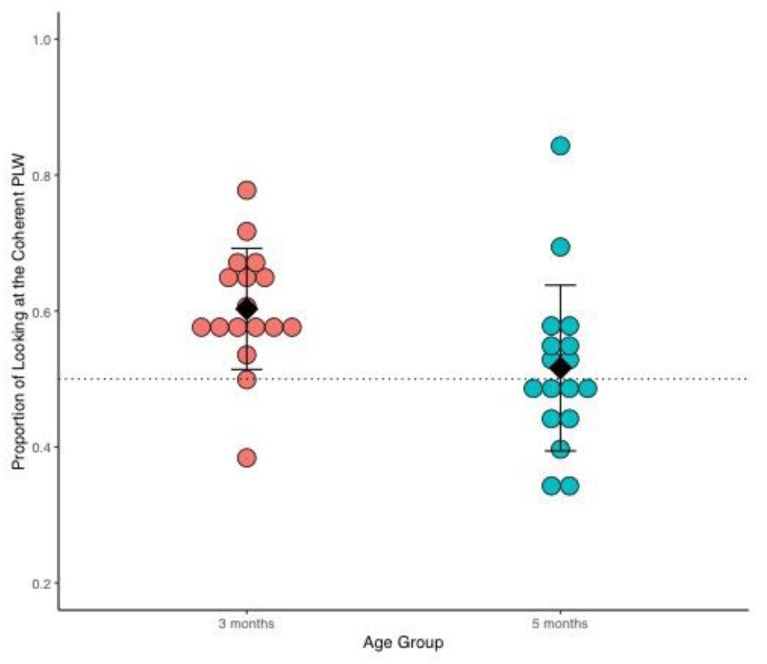
Proportion of looking at the coherent point-light walker (PLW) in Experiment 1 at three and five months of age. Each dot represents the proportion of looking at the coherent point-light walker for an individual subject. Three-month-old infants are denoted by orange dots (*n* = 17), and blue dots denote five-month-old infants (*n* = 17). Mean group proportion per age group is represented by the black diamonds and the black bars denote 1 +/− standard deviation of the mean. The doted horizontal line marks the 0.50 proportion, (i.e., equal looking at both stimuli) and no preference.

Results from our first experiment revealed that three-month-old infants looked longer at the coherent point-light walker (M = 173.70 s, SD = 42.12) in relation to the scrambled point-light walker (M = 111.59 s, SD = 28.69), whereas this difference was not evident in the amount of time that five-month-old infants spent looking at the coherent (M = 128.46 s, SD = 123.34) and scrambled (M = 123.34 s, SD = 39.44) point-light walkers—see Figure 2. The looking time data for each participant are also available in Table S1 of the Supplementary Materials.

To determine a preference for the coherent point-walker in the two age groups, we compared the grand mean proportion of time looking at the coherent point-light walker to chance level (0.50). A proportion of looking at the coherent point-light walker that is not statistically different from 0.50 indicates no preference for either stimulus; a proportion of looking at the coherent point-light walker that is significantly above 0.50 indicates a preference for the coherent point-light walker. Finally, to determine if the two groups statistically differ in their preferences, we also compared the grand mean proportion of looking at the coherent point-light walker between the two age groups.

#### 2.2.1. Comparison to Chance Level

Three-month-old infants preferred to look at the coherent point-light walker: the grand mean proportion of looking at the coherent point-light walker, M = 0.60 (60%), SD = 0.08, is significantly above chance, *t*(16) = 4.77, *p* < 0.001. As can be seen in Figure 2, the proportion of looking at the coherent stimulus is above 0.50 in all subjects at this age, except for two infants.

In contrast, as a group, five-month-old infants did not prefer to look at the coherent point-light walker, M = 0.52 (52%), SD = 0.12, *t*(16) = 0.55, *p* = 0.59. Infants at this age do not show a systematic preference for the coherent or the scrambled point-light walker—see five-month-old data in Figure 2.

#### 2.2.2. Comparison between Age Groups

The three and five-month-old infants’ preferences significantly differed, with a grand mean proportion of looking at the coherent point-light walker being significantly higher at three when compared to five months, *t*(32) = 2.38, *p* = 0.02.

### 2.3. Discussion

We intended to find when, in infancy, a preference for a coherent point-light walker over a scrambled walker emerges. Results from our first experiment enable us to answer our main research question: a preference for biological motion configuration is present at least by three months of age. Nevertheless, five-month-old infants did not show any systematic preference.

#### 2.3.1. Three-Month-Old Infants Prefer the Coherent Point-Light Walker

Previous preferential looking experiments report that newborns show no preferences between a coherent and a scrambled point-light walker [6]. In contrast with newborns, three-month-old infants in our sample showed a significant proportion of looking at a coherent point-light walker in relation to a scrambled control. Our observation of a preference at three months adds further evidence by indicating that a first transition to a preference for biological motion configuration happens in our first three months of life.

Importantly, a preference for the intact display does not necessarily mean that the global human motion configuration of the walker is being processed at three months—previous habituation experiments suggest that three-month-old infants are only tuned to local (and not to global) configural information in point-light walkers [17,21,22,41]. Thus, we hypothesize that our measured preference is due to the ability in these infants to perceive only local configural information [17]. The scrambled point-light walker has all configural relations between the dots removed; however, both global and local configural information can be extracted from the coherent walker. Three-month-old infants in our sample might prefer the coherent walker because they perceived local configural information (also only available in the coherent display). This interpretation is in line with previous habituation experiments conducted with three- and five-month-old infants that show that younger infants are more tuned to local configural information, while five-month-old infants, to the global configuration of a person walking [17]. Either way, our results indicate that perceiving configural information is key in determining three-month-old infants’ preferences to the coherent and relevant motion of a person walking. This behavior is important, as it likely has cascading effects in infants’ motor and social–cognitive development later in infancy [42,43,44,45,46].

At last, it is worth mentioning that, considering the nature of the preferential looking procedure [47,48], and because three-month-old exhibited a preference, we can also infer that three-month-old infants in our study discriminate between a coherent and a scrambled point-light walker; to prefer one stimulus, infants have to discriminate between the two stimuli.

#### 2.3.2. Five-Month-Old Infants Reveal No Systematic Preferences

In our first experiment, five-month-old infants revealed no systematic preference for neither coherent nor scrambled displays; this result is unexpected for two main reasons: first, because previous habituation experiments report that five-month-olds discriminate between a coherent and a scrambled point-light walker [17,18,19], indicating that five-month-olds are sensitive to the differences between the two stimuli—thus, it would make sense that they would exhibit a preference; second, because previous preferential looking experiments report a preference for upright biological motion stimuli (intact displays vs. inverted controls) in three- to six-month-old infants [34,35]. In particular, Sifre et al. (2018) found that preferences for upright biological motion emerge at three months and become increasingly pronounced during infancy in subsequent months. In contrast, we found a decrement in preference for the coherent display with developmental age.

Thus, we conjectured that our measured decline in preference could be explained by some parameter of the procedure that could be not so well adjusted to the older infants. In particular, we conjecture that 60 s of trial duration (what we used with both three- and five-months old) might be too long for five-month-olds.

Trial duration in preferential looking experiments with biological motion stimuli varies greatly across the literature: while some studies conducted with newborns [7,33,49] and older infants [34] employed 60 s of trial duration, other studies with four- to twelve-month-old infants employed less time, varying between 10.5 s [50,51] and 15–20 s [35,38,52]. To understand if 60 s is too long for the older infants, and to assess more information regarding the procedure in both ages, we decided to analyze graphically the individual temporal looking patterns of the two age groups, per trial—see Figures S1 and S2 of the Supplementary Materials for a visualization of the looking patterns, per infant in the two age groups. Five-month-olds show more transitions in terms of exhibiting several short looks in the two stimuli (or an increased number of transitions between stimuli), and three-month-old exhibit fewer looks but longer looking bouts. To further compare these differences, we calculated and compared the number of looking bouts per minute in the two age groups during the experiment, as well as the total looking time at both screens in the two age groups [53]. Five-month-old infants (mean number of transitions per minute = 14.02, SD = 4.11) differed from three-month-old (M = 9.96, SD = 5.28) because they significantly produced more transitions than the younger infants, *t*(32) = 2.50, *p* = 0.02. The two groups also differed in their proportion of total looking time to the screens, *t*(32) = 2.30, *p* = 0.03, with five-month-old infants (proportion of total looking time: 0.71, SD = 0.12) spending less time looking at the screens than the younger infants (M = 0.81, SD = 0.12), though infants at both age-groups were measurably engaged in the task since they had to look at least 50% of the time to either screen. It appears that the coherent point-light walker was grabbing and holding the three-month-old infants’ attention, while at five months, the two stimuli were effective at grabbing the infants’ attention but not in holding their attention for longer periods, indicating that 60 s is perhaps too long for five-month-old infants [53]. Trial duration is a critical parameter that must be adapted both to infants’ age and infants’ speed of encoding the stimuli—older infants tend to encode visual stimuli faster than younger infants [47]. Therefore, it makes sense that the older five-month-old infants need less time for stimuli presentation.

Considering the exposed, we decided to conduct a second preferential-looking experiment with the goal of replicating our main finding with five-month-olds but using a reduced trial duration. We also wondered if this difference in performance (preference at three vs. no preference at five) is specific to five-month-olds or if it is present at older age groups, likely reflecting the developmental shift previously reported between three and five months of age [17]. Thus, in this second experiment, we also included a new age group of seven-month-old infants.

In Experiment 2, we asked parents that participated in the first experiment with their infants at three months to return both at five and at seven months of age. This second experiment is longitudinal.

## 3. Experiment 2: Longitudinal Study with 3-, 5- and 7-Month-Old Infants

### 3.1. Methods

#### 3.1.1. Participants

We contacted parents that participated in the first experiment when their infant was three months in age to come again to participate at five (mean age = 171 days, SD = 12.42, age range = 155.0–193.0 days, 6 females) and seven months (mean age = 228.75 days, SD = 11.29, age range = 212.0–254 days, 8 females), except for one infant that started participating at five months. Thus, *n* = 28 infants were enrolled in this study: 27 infants participated since the beginning at three months, and one infant began participation at five months and returned at seven months. To be included on final sample, an infant had to have usable data in at least two time points.

The final longitudinal sample consisted of *n* = 17 infants: *n* = 14, three-month-olds; *n* = 14, five-month-olds; and *n* = 16, seven-month-olds. From the first to the second experiment, three subjects were excluded from the final sample at three months of age because (1) parents did not come to participate again (*n* = 2), and (2) one subject did not have usable data in any of the other two time-points. However, two infants that participated at three months of age in Experiment 1 (but were excluded due to the two looking criteria at this time point) were now included since they had usable data both at five and seven months. Plus, at five months of age, one infant missed the data collection session, and two were eliminated due to technical problems. Only one infant missed participation at seven months of age.

All parents signed the consent form, and the University of Minho ethics’ committee approved the study.

#### 3.1.2. Stimuli

We used the same stimuli as in Experiment 1.

#### 3.1.3. Procedure

The same procedure applied to Experiment 1, except that trial duration for infants at five months was reduced from 60 s to 20 s, and infants participated in a total of 12 trials instead of 6. At seven months of age, each infant participated in 12 trials of 15 s each.

#### 3.1.4. Data Coding and Processing

We applied same processing done for Experiment 1. To be included in the final sample, infants had to pass the same two looking criteria as in Experiment 1.

In addition, only infants with usable data from at least two time-points were included in our longitudinal sample. Looking time to the coherent point-light walker was again calculated per trial and transformed into a proportion. This proportion was then averaged per subject. Proportion data followed a normal distribution.

### 3.2. Results

As in Experiment 1, three-month-old infants spent more time looking at the coherent point-light walker (Mean total looking time = 178.53 s, SD = 44.68) in relation to the scrambled point-light walker (M = 116.54 s, SD = 30.30) but not five-(M coherent = 82.80 s, SD = 16.81; M scrambled = 76.07, SD = 15.18) and seven-month-old infants (M coherent = 60.11 s, SD =; M scrambled = 55.11 SD = 14.24); these infants looked the overall same amount of time at both stimuli. The looking time data for each participant are available in Table S2 of the Supplementary Materials.

**Figure 3:**
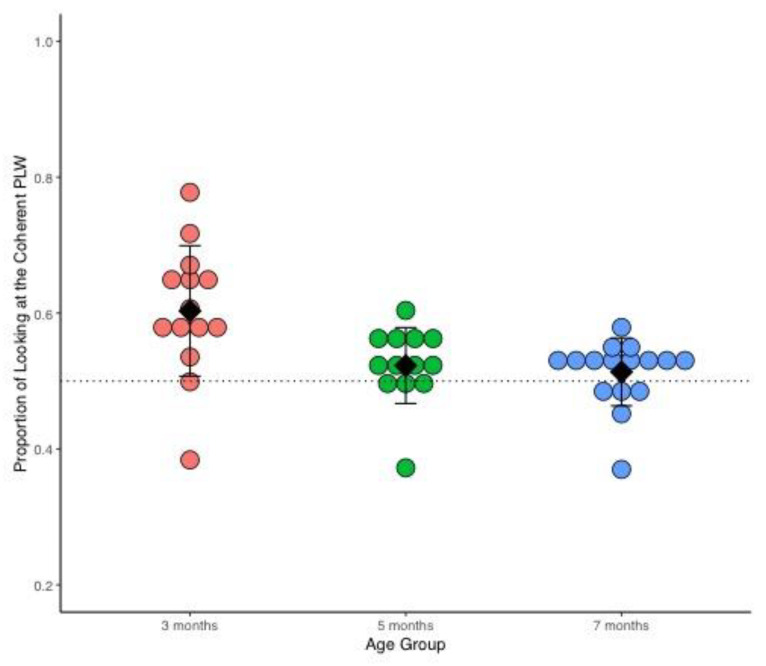
Proportion of looking at the coherent point-light walker (PLW) in Experiment 2 at three (red), five (green) and seven (blue) months of age. The data at three months of age are the same as the three-month-old group in Experiment 1. Each dot represents the proportion of looking at the coherent point-light walker per subject. Infants at three months of age are denoted by orange dots (*n* = 14), green dots denote five months (*n* = 14), and blue seven-month-olds (*n* = 16). Mean group proportion per age group is represented by the black diamonds, and the black bars denote 1 +/− standard deviation of the mean. The doted horizontal line marks the 0.50 proportion, (i.e., equal looking at both stimuli) and no preference.

The main finding of this experiment is evident in Figure 3: the grand mean proportion of looking at the coherent point-light walker at five and seven months of age is close to chance level, M = 0.52, SD = 0.05, and M = 0.50, SD = 0.05 respectively. In contrast, the grand mean proportion at three months of age is above chance, M = 0.60, SD = 0.09.

Visual comparison of Figure 2 and Figure 3 also suggests that the variance in proportion of looking at the coherent point-light walker at five months is greater in Experiment 1 in relation to this second experiment. In other words, the introduction of a shorter trial duration seems to have reduced the variance of the data at five months in this second experiment.

**Figure 4:**
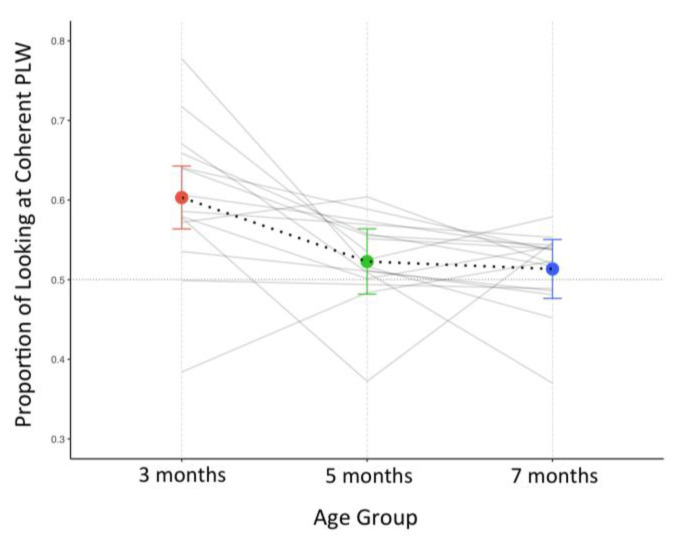
Model-based mean estimates of proportion of looking at the coherent point-light walker at three (orange), five (green) and seven (blue) months of age superimposed onto infants’ longitudinal raw data (in grey). The horizontal dotted line denotes the 0.5 proportion, (i.e., equal looking at both stimuli) and no preference. Error bars are displayed through mean dots.

We fitted our longitudinal data for mean proportion of looking at the coherent point-light walker, with a linear mixed-effect model. Data were modeled using the nlme package in R [54] using age in months (3 levels) as a fixed effect and a subject-level intercept as a random effect—see Figure 4 to see the model estimates over the three time points.

To determine a preference for the coherent point-light walker, we first compared the model’s estimated mean proportion in the three age groups against the 0.50 chance level. We tested whether the mean proportion of looking at the coherent point-light walker in three-, seven- and five-month-old infants was different from chance by computing a 95% confidence interval of the model’s estimated mean proportion and a single tailed test (adjusted for multiple comparisons using the Bonferroni correction) against 0.50. The confidence interval and test were obtained using the R package emmeans [55]. Secondly, we compared the estimated mean proportion of looking at the coherent point-light walker in the three age groups.

Lastly, and to understand the effect of changes in trial duration in the two five-month-old samples (Experiment 1 vs. Experiment 2), we compared mean proportion of looking at the coherent point-light walker at five months in Experiment 1 and Experiment 2. Then, we further compared variance of proportion of looking at the coherent point-light walker in the two five-month-old samples.

#### 3.2.1. Comparison to Chance Level

Mean estimates from the model confirmed preferential attention to the coherent point-light walker at three months of age but absent at five and seven months; mean proportion of looking at the coherent point-light walker was significantly above chance at three months, *t*(16) = 5.54, *p* < 0.001, but not at five *t*(16) = 1.18, *p* = 0.26, or seven months, *t*(16) = 0.77, *p* = 0.45.

#### 3.2.2. Comparison between the Three Age Groups

We also compared the estimates of the mixed model between the three age groups: proportion of looking at the coherent point-light walker was significantly greater at three months both when comparing with five, *t*(24) = 2.99, *p* < 0.01, and seven-month-old infants, *t*(24) = 3.52, *p* < 0.05. Differences between five and seven months were not significant, *t*(24) = 0.026, *p* = 1.00.

#### 3.2.3. Comparison of Five-Month-Old Infants’ Looking Data in Experiment 1 and Experiment 2

We compared the mean proportion of looking at the coherent point-light walker at five months in Experiment 1 to Experiment 2 (two independent samples with equal variances not assumed): no significant differences were found between the two samples, *t*(22.864) = −0.189, *p* = 0.85.

Finally, we also compared variance in the proportion of looking at the coherent point-light walker in Experiment 1 (M = 0.52, SD = 0.12) to Experiment 2 (M = 0.52, SD = 0.05). We computed an F test comparing the two variances and found that variance was significantly larger in the Experiment 1 in relation to Experiment 2 at five months, *F*(16,13) = 5.15, *p* < 0.01.

### 3.3. Discussion

Experiment 2 was important because we replicated the same result found on Experiment 1 at five months, using a reduced trial duration (i.e., no preference for biological motion configuration), and we extended this result to seven-month-old infants.

Younger three-month-old show a clear preference for the coherent point-light walker: three-month-old infants perceive and orient to biological motion configuration (possibly due to the processing of local configural information also only present in the coherent display).

Altogether, our results reveal an important developmental shift between three and five months of age in infants’ preferences for biological motion [17].

#### 3.3.1. Five-Month-Old Infants Reveal No Systematic Preferences Both in Experiment 1 and 2

Five-month-old infants do not prefer a coherent walker in relation to a scrambled point-light walker: we found this result in two preferential looking experiments, conducted with two independent samples of infants. From the first to the second experiment, we reduced trial duration from 60 to 20 s; the fact that we still found no preferences in Experiment 2 demonstrates that our result at five months is not explained by an inappropriate trial duration in the preferential looking procedure. Importantly, the introduction of a shorter trial duration reduced the variance in the proportion of looking at the coherent display, from Experiment 1 to Experiment 2. We can thus conclude that 20 s of trial duration is a more adequate parameter for the five-month-old infants in this task. This makes sense since the older the infants, the faster they tend to encode visual stimuli [47]. Five-month-olds need less exposure than three-month-olds to process point-light walkers in a preferential looking task.

#### 3.3.2. Decline in Preference for the Coherent Point-Light Walker in the Older Infants

Five- and seven-month-old infants did not show any systematic preference in Experiment 2. This response seems to be at odds both with neuroimaging findings with infants and point-light displays [23,24,25,26,56], and with the few preferential looking experiments conducted so far with point-light stimuli [34,35].

Sifre et al. (2018) tested longitudinally infants from 2 to 24 months of age in their preferences toward intact upright point-light displays of an actor playing children’s games, and inverted versions of the displays. They found a preference for the upright displays emerging at three months and increasing across developmental age [34]. Like Sifre et al. (2018), we also found a preference for biological motion configuration at three months but followed by a decline in preference for the coherent display through developmental age. Our study, and that of Sifre at al. (2018) have several differences that might account for these discrepant results. First, Sifre et al. (2018) used multimodal stimuli (i.e., point-light displays were presented with audio recordings of the actions) and employed displays of different and complex actions, while we applied the visual-only stimulus of a walker. However, the main difference here is that Sifre et al. (2018) used an inverted control and not a scrambled display. An inverted point-light display has configural coherence (although inverted), thus both local and global coherent configurations, and the individual motions of the dots are inverted. Our scrambled point-light walker has no figural coherence, but the individual motions of the dots are intact. Therefore, a preference for an upright point-light walker in relation to an inverted one tell us less about infants’ configural processing ability in biological motion perception, and likely more about infants’ sensitivity to the gravity-dependent dynamics of motion.

ERP [23] and fNIRS studies [25] report differential brain responses to coherent and scrambled displays in infants. Lisboa, Queirós, et al. (2020) measured responses in seven-month-old infants using fNIRS with the exact same point-light stimuli that we present here. Results from this neuroimaging experiment suggest a mature-like processing of biological motion configuration at seven months: only the coherent point-light walker activated the right middle-posterior temporal cortex, an area known for its role on human social–cognitive perception and involved in adults’ mature processing of biological motion configuration [27,28,29,30,31,32]. This result indicates that there is a cortical response specific to the presence of biological motion configuration, and hence, it would make sense that these infants would turn their attention the coherent configuration of a person walking. However, this was not the case in our study. Next, we argue that this absence of a preference can be explained by multiple factors and does not necessarily mean that infants are not able to process configural relations, or that the underlying cortical mechanisms are not functioning [25,57]. Our results do support the existence of a developmental shift in infants’ preferences for biological motion configuration, happening between three and five months of age.

#### 3.3.3. Developmental Shift between 3 and 5 Months of Age in Biological Motion Perception

We identified a developmental shift in infants’ preferences for biological motion configuration between three and five months of age: three-month-olds preferred the coherent point-light walker in relation to the scrambled point-light walker whereas both five and seven-month-old showed no preferences. This shift is, nevertheless, unexpected [25,34]. Considering only the older infants’ looking behavior and the simplest interpretation of a preferential looking task, we found no evidence for the ability to process configural relations in human motion both at five and seven months of age. Simple looking time measures provide very limited “yes/no” information about infants’ social cognition. There can be many possible interpretations for the lack of preference that does not necessarily mean that older infants are not able to process biological motion configuration. We discuss three speculative interpretations (not mutually exclusive) for this shift in preference.

In our first hypothesis, we consider that different attentional processes in the two age groups could have mediated the differential preferential responses to the stimuli in the task. Three-month-old attention is more exogenous or stimulus driven, that is, more controlled by the external characteristics of the stimuli; however, at five months, infants gain a more endogenous control of their attention and are internally directed [58,59]. Thus, it could be that three months’ selective attention in our study was driven by the “low-level” visual features of the coherent point-light walker (such as the depiction of configural coherence or a vertical symmetry), whereas the older infants, likely less controlled by these salient visual features of the coherent stimulus, engaged in a more “exploratory” looking behavior, thus, switching their looks between the two stimuli—see Figures S1 and S2 of the Supplementary Materials for a visualization of the looking patterns of infants at the two age groups.

Our second hypothesis refers to the level of complexity of the two stimuli. The scrambled point-light walker is visually more complex than the coherent walker. Bertenthal, Proffitt, Kramer et al. (1987) reported, in their infant-controlled habituation experiment, that three-month-olds took longer to habituate to the scrambled in relation to the coherent point-light walker. This result suggests that the scrambled walker is visually more complex for these infants since they need more time to encode it [19]. We know that infants do not prefer stimuli that are either too simple or too complex and prefer stimuli with intermediate levels of complexity [60]. In this sense, three-month-old infants might have turned their attention to the stimulus with an intermediate level of complexity for their visual system to process, that is, the coherent display. In contrast, the scrambled point-light walker might be a visual competitor of more comparable complexity to the coherent walker for the older infants; therefore, five- and seven-month-olds equally turn at both stimuli.

The final and third hypothesis considers that a preference for the coherent point-light walker might emerge later in development in a *U*-shape developmental curve and linked to infants’ motor development. The infants’ own action experience seems to be related to the infants’ visual preferences: crawling infants preferred to look at a crawling motion and walking infants prefer a walking motion [52]; ten-week-old infants, who experienced the stepping reflex, produced greater ERP activity when viewing an upright coherent point-light walker relative to an inverted walking motion—this effect was not found in infants that did not experience the walking reflex [61]. Thus, we conjecture that a preference for the motion of a person walking in relation to a scrambled control might emerge later in development, at around 14–19 months of age in experienced walking infants.

## 4. Final Conclusions and Future Directions

We conducted two preferential looking experiments measuring preferences for human walking motion configuration in the first half of infants’ first year of life. We found a preference for the coherent motion of a person walking at three months but a subsequent decline (or no preference) both at five and at seven months of age. Multiple reasons might explain this decline in preference for the coherent walker; we have identified and discussed three hypotheses, namely, a different balance between exogenous and endogenous selective attention at the two age groups; a different effect of the stimulus complexity at the two age points; and the different motor development of the infants that could impact their preferences. None of these interpretations implicate older infants’ inability to perceive the global configuration of the human walking action. Moreover, we hypothesize that three-month-olds’ preferences for the coherent display are explained by an ability of these infants to perceive local (but not global) configural information in biological motion. Younger infants’ abilities could be investigated by testing three-month-old infants in the same preferential looking procedure but contrasting a coherent point-light walker with a set of moving limbs randomly displayed (i.e., with a control stimulus in which local configural information is intact but with no human global configuration). We hypothesize that, in this latter case, the preference for the coherent walker at three months will dissipate.

In this context, it is critical to consider the limitations of the method (preferential looking) as well as the importance of having convergent measures for determining infants’ abilities. The preferential looking procedure is a classical method from developmental psychology that has been extensively used to investigate infants’ social cognition, especially considering its simplicity and easy implementation with infants. However, its main limitation is that it bases its conclusions on a very limited response (i.e., in looking [62]). In the case of our studies, we intended to find the relation between infants’ ability to perceive configural relations in biological motion; its interpretation has a relevant stimulus that they would actively turn their attention or prefer. Nevertheless, considering our ambiguous findings with the older infants, more studies with different techniques and more convergent variables are needed to disambiguate our findings, overcome some of the procedures’ limitations, and increase our understanding of biological motion perception in infancy. For instance, using an eye tracker in our design would add information about the way infants scan or perceive the two stimuli; other methods could include the infant’ habituation procedure or neuroimaging methods. Future studies could also consider the particular social and motor development of the infant [51], as chronological age is only one possible developmental descriptor but might not always be the best one.

The studies presented here are relevant since they bring to the discussion how the study of basic perceptual phenomena is important and contributes to a better comprehension of how humans make sense of actions.

## Supplementary Materials

The following supporting information can be downloaded at: https://www.mdpi.com/article/10.3390/brainsci12050566/s1, Figure S1: Visualization of 3-month-olds’ looking time series in Experiment 1; Figure S2: Visualization of 5-month-olds’ looking time series in Experiment 1. Table S1: Total looking time and mean proportion of looking at the coherent and scrambled point-light walkers (PLWs) per subject (final sample) and age group in Experiment 1 (cross-sectional). Table S2: Total looking time and mean proportion of looking at the coherent and scrambled point-light walkers (PLWs) per subject (final sample) and age group in Experiment 2 (longitudinal).
