# Supplementary material for: Three Months-Old’ Preferences for Biological Motion Configuration and Its Subsequent Decline
Source: Brain Sci. 2022 Apr 27;12(5):566. doi: 10.3390/brainsci12050566 (PMC9139228; doi:10.3390/brainsci12050566)

Figure S1. Visualization of three-month-olds' looking time series data in Experiment 1. Each row of colored tiles corresponds to one individual infant and each horizontally contiguous block of color corresponds to a sustained bout of fixation towards the coherent PLW, the scrambled PLW or Away. Trial duration was 60s and the start of each trial was synchronized in the visualization. The vertical ordering of infants was calculated by applying hierarchical clustering (Ward's method) to the vector composed of proportion of looking at coherent PLW per trial. A gap between trials was placed to help the visualization; inter-trial pause took only a few seconds and was controlled by the infant's behavior.

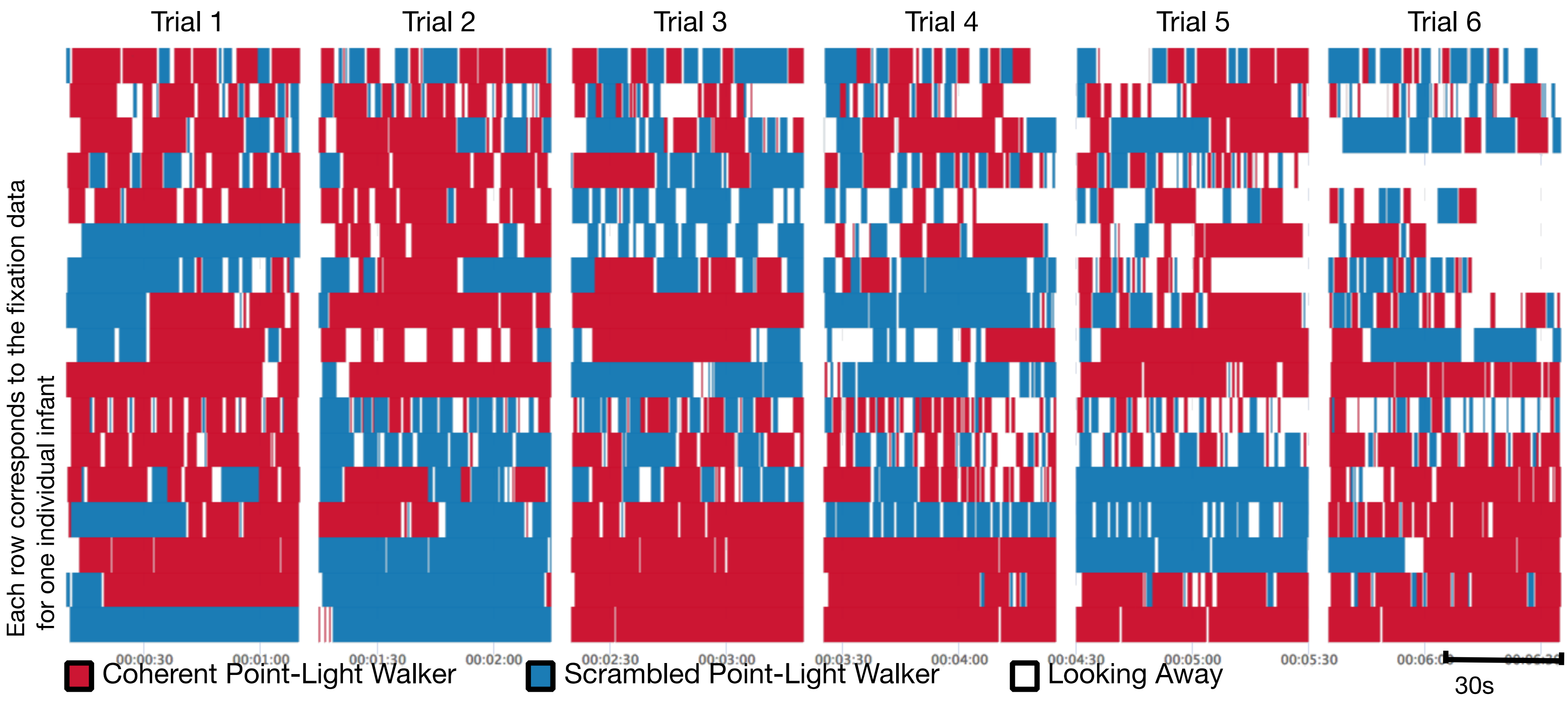

Figure S2. Visualization of five-month-olds' looking time series data in Experiment 1. Each row of colored tiles corresponds to one individual infant and each horizontally contiguous block of color corresponds to a sustained bout of fixation towards the Coherent PLW, the Scrambled PLW or Away. Trial duration was 60s and the start of each trial was synchronized in the visualization. The vertical ordering of infants was calculated by applying hierarchical clustering (Ward's method) to the vector composed of proportion of looking at Coherent PLW per trial. A gap between trials was placed to help the visualization; inter-trial pause took only a few seconds and was controlled by the infant's behavior.

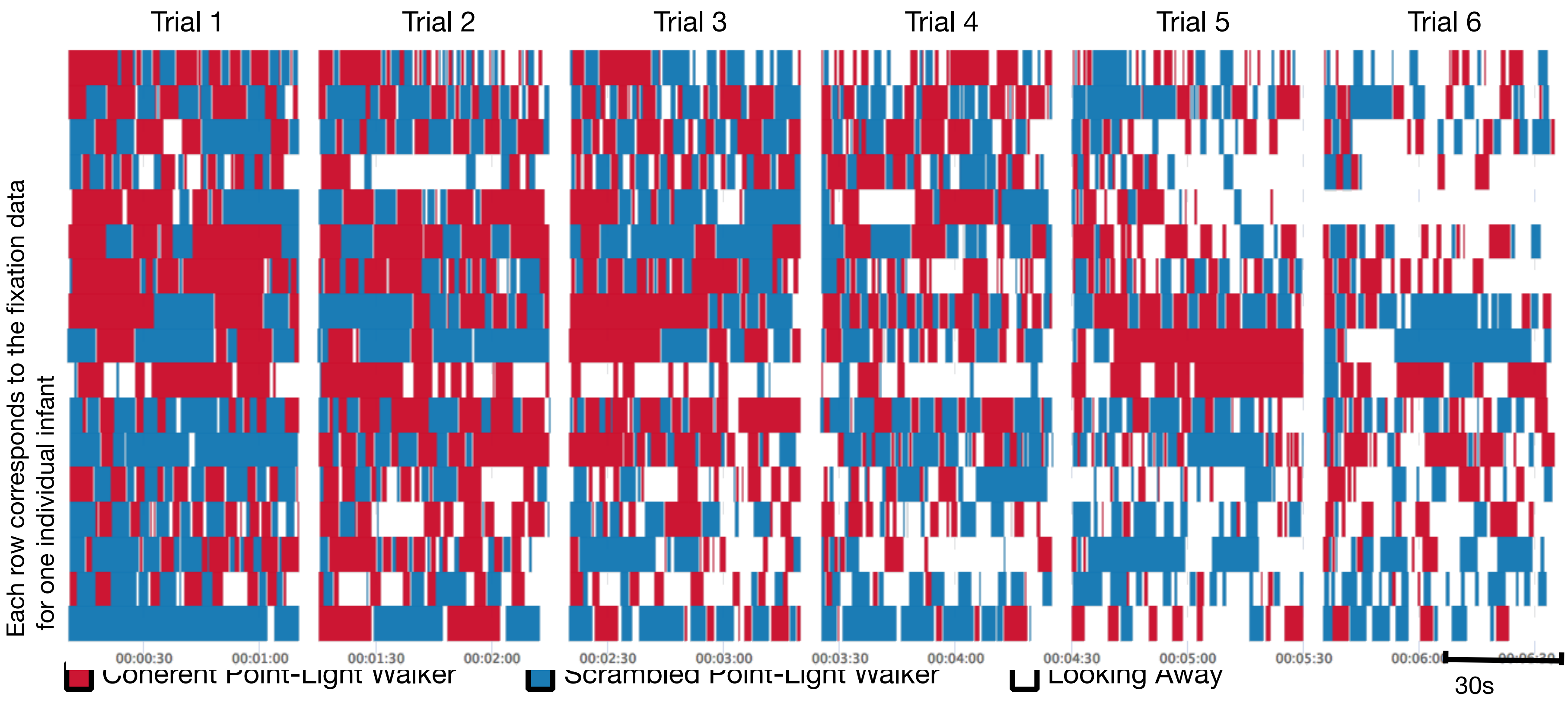

Supplement: Supplementary file 1 [file brainsci-12-00566-s001.zip › Fixation Time Series Graphs MUVTIME.pdf]
