# Supplementary material for: Three Months-Old’ Preferences for Biological Motion Configuration and Its Subsequent Decline
Source: Brain Sci. 2022 Apr 27;12(5):566. doi: 10.3390/brainsci12050566 (PMC9139228; doi:10.3390/brainsci12050566)
Supplement: Supplementary file 1 [file brainsci-12-00566-s001.zip › Tables S1&S2.pdf]

Table S1. Total looking time and mean proportion of looking at the coherent and scrambled point-light walkers (PLWs) per subject (final sample) and age group in Experiment 1 (cross-sectional).

		Total Looking time (in secs)		Mean Proportion of Looking	
Age (in months)	Subject ID	Coherent PLW	Scrambled PLW	Coherent PLW	Scrambled PLW
3	1	155.89	85.73	0.65	0.36
	2	134.09	79.53	0.63	0.43
	3	129.13	107.95	0.54	0.41
	4	128.72	93.23	0.58	0.43
	5	173.56	138.02	0.56	0.42
	6	154.54	128.87	0.55	0.46
	7	208.93	136.12	0.61	0.39
	8	237.96	115.26	0.67	0.33
	9	165.41	155.01	0.52	0.50
	10	262.68	77.3	0.77	0.22
	11	89.48	170.2	0.34	0.62
	12	218.62	107.7	0.67	0.34
	13	165.4	114.02	0.59	0.42
	14	159.73	55.62	0.74	0.28
	15	203.26	110.07	0.65	0.36
	16	174.86	129.65	0.57	0.43
	17	190.67	92.68	0.67	0.33
5	1	125.75	97.03	0.56	0.42
	2	162.54	125.05	0.57	0.42
	3	145.77	156.92	0.48	0.53
	4	90.45	143.39	0.39	0.6
	5	177.26	20.99	0.89	0.16
	6	104.01	105.01	0.50	0.5
	7	110.12	99.6	0.53	0.45
	8	168.53	154.57	0.52	0.48
	9	65.41	125.57	0.34	0.66
	10	129.42	101.4	0.56	0.45
	11	130.5	161.04	0.45	0.56
	12	140.12	153.02	0.48	0.51
	13	128.19	129.62	0.50	0.50
	14	143.55	169.58	0.46	0.56
	15	179.81	80.07	0.69	0.31
	16	81.79	185.21	0.31	0.65
	17	100.6	88.73	0.53	0.46

Table S2. Total looking time and mean proportion of looking at the coherent and scrambled point-light walkers (PLWs) per subject (final sample) and age group in Experiment 2 (longitudinal).

		Total Looking Time (in secs)		Mean Proportion of Looking	
Age (in months)	Subject ID	Coherent PLW	Scrambled PLW	Coherent PLW	Scrambled PLW
3	1	155.89	85.73	0.65	0.35
	19	129.13	107.95	0.54	0.46
	21	173.56	138.02	0.56	0.44
	26	154.54	128.87	0.55	0.45
	28	208.93	136.12	0.61	0.39
	30	237.96	115.26	0.67	0.33
	32	165.41	155.01	0.52	0.48
	41	262.68	77.30	0.77	0.23
	54	89.48	170.20	0.34	0.66
	55	218.62	107.70	0.67	0.33
	56	165.40	114.02	0.59	0.41
	57	159.73	55.62	0.74	0.26
	60	203.26	110.07	0.65	0.35
	73	174.86	129.65	0.57	0.43
5	21	74.48	113.64	0.40	0.60
	28	106.41	82.00	0.56	0.44
	30	59.61	64.01	0.48	0.52
	34	96.53	88.83	0.52	0.48
	41	91.20	84.42	0.52	0.48
	42	106.22	87.50	0.55	0.45
	54	65.70	74.28	0.47	0.53
	55	73.01	55.85	0.57	0.43
	56	89.06	88.15	0.50	0.50
	57	71.95	60.24	0.54	0.46
	58	66.05	62.06	0.52	0.48
	60	71.67	56.99	0.56	0.44
	73	104.49	70.92	0.60	0.40
	1	67.07	58.97	0.53	0.47
7	19	82.73	68.84	0.55	0.45
	21	67.38	56.91	0.54	0.46
	26	17.77	21.57	0.45	0.55
	28	56.28	45.55	0.55	0.45

	30	43.25	63.98	0.40	0.60
	32	63.22	67.90	0.48	0.52
	34	58.20	61.47	0.49	0.51
	41	62.59	74.32	0.46	0.54
	42	68.14	61.34	0.53	0.47
	54	52.04	41.33	0.56	0.44
	55	49.70	40.47	0.55	0.45
	56	76.87	64.18	0.54	0.46
	58	57.28	39.46	0.59	0.41
	60	66.21	48.16	0.58	0.42
	73	73.11	67.30	0.52	0.48
